# Assessment of a Multifaceted Approach, Including Frequent PCR Testing, to Mitigation of COVID-19 Transmission at a Residential Historically Black University

**DOI:** 10.1001/jamanetworkopen.2021.37189

**Published:** 2021-12-13

**Authors:** Neil G. Hockstein, LaKresha Moultrie, Michelle Fisher, R. Christopher Mason, Derrick C. Scott, Joan F. Coker, Autumn Tuxward, Juliana Terheyden, Nolan Canter, Michael Coons, Saundra DeLauder, Tony Allen

**Affiliations:** 1 Testing for America; 2 Department of Surgery, ChristianaCare, Newark, Delaware; 3 ENT and Allergy of Delaware, Newark; 4 Delaware State University, Global Institute for Equity, Inclusion, and Civil Rights, Dover; 5 Office of General Counsel, Delaware State University, Dover; 6 Campus Health Services, Delaware State University, Dover; 7 Department of Public and Allied Health, Delaware State University, Dover; 8 Department of Biological Sciences, Delaware State University, Dover; 9 Molecular Diagnostics Laboratory, Delaware State University, Dover; 10 New Castle County Delaware Department of Emergency Medical Services, New Castle; 11 Division of Academic Affairs, Delaware State University, Dover; 12 Office of the President, Delaware State University, Dover, Delaware

## Abstract

**Importance:**

COVID-19 posed an unprecedented threat to residential colleges in the fall of 2020. While there were mathematical models of COVID-19 transmission, there were no established or tested protocols of COVID-19 testing or mitigation for school administrators to follow.

**Objective:**

To investigate the association of a multifaceted COVID-19 mitigation strategy using social, behavioral, and educational interventions and a program of frequent testing with prevalence of disease spread.

**Design, Setting, and Participants:**

This cohort study was conducted as a retrospective review of COVID-19 positivity from August 16, 2020, to April 30, 2021, at Delaware State University, a publicly funded historically Black university. Participants included all students, faculty, and staff members with a campus presence. Positivity rates after use of mitigation strategies and testing on campus were compared with those of the surrounding community. Data were analyzed from July through September 2021.

**Exposures:**

Mitigation strategies included education and outreach about social distancing, masking, and handwashing, and a COVID-19 testing plan consisted of twice-weekly polymerase chain reaction (PCR) screening using anterior nasal samples (fall and early spring semester) and then saliva-based samples (middle to late spring semester).

**Main Outcomes and Measures:**

Cumulative tests, infections, daily quarantine, and isolation residence hall occupancy were measured, and comparisons were made with statewide COVID-19 positivity rates.

**Results:**

The campus cohort included 2320 individuals (1575 resident students, 415 nonresident students, and 330 faculty or staff members). There were 1488 (64.1%) women and 832 (35.9%) men; mean (SD) age was 27.5 (12.9) years. During the fall semester, 36 500 COVID-19 PCR tests were performed. Weekly positivity rates ranged from 0 of 372 tests to 16 of 869 tests (1.8%) (mean [SD] positivity rate, 0.5% [0.5%]; 168 positive results and 36 312 negative results). During the same period, statewide positivity ranged from 589 of 25 120 tests (2.3%) to 5405 of 54 596 tests (9.9%) (mean [SD] positivity rate, 4.8% [2.6%]). In the spring semester, 39 045 PCR tests were performed. Weekly positivity rates ranged from 4 of 2028 tests (0.2%) to 36 of 900 tests (4.0%) (mean [SD] positivity rate, 0.8% [0.9%]; 267 positive results and 38 767 negative results). During the same period, statewide positivity ranged from 1336 of 37 254 tests (3.6%) to 3630 of 42 458 tests (8.5%) (mean [SD] positivity rate, 5.1% [1.3%]). Compared with statewide rates, campus positivity rates were mean (SD) 4.4 (2.6) percentage points lower during the fall semester (*P* < .001) and mean (SD) 5.6 (1.6) percentage points lower during the spring semester (*P* < .001). Total daily quarantine and isolation residence hall occupancy ranged from 0 to 43 students in the fall and 1 to 47 students during the spring.

**Conclusions and Relevance:**

This study found that the combination of campuswide mitigation policies and twice-weekly COVID-19 PCR screening was associated with a significant decrease in COVID-19 positivity at a residential historically Black university campus compared with the surrounding community. Given the socioeconomic demographics of many students at historically Black colleges and universities, keeping these resident campuses open is critical not only to ensure access to educational resources, but also to provide housing and food security.

## Key Points

**Question:**

Would a multimodal strategy of mitigation and testing be adequate to safely reopen a residential historically Black college and university (HBCU) campus in the setting of increasing COVID-19 positivity rates?

**Findings:**

In this cohort study among 2320 individuals at a publicly funded HBCU, combining an active messaging campaign on shared responsibility and mitigation measures with frequent polymerase chain reaction testing was associated with decreased COVID-19 positivity rates compared with the surrounding community. The need for isolation and quarantine dormitory occupancy was limited.

**Meaning:**

This study found that, in the setting of increased community COVID-19 positivity rates and the absence of a vaccine, well-described mitigation efforts and frequent testing were associated with decreased spread of disease.

## Teaser

This cohort study examines the association of mitigation and testing strategies with COVID-19 positivity rates during reopening of a historically Black university during the pandemic.

## Introduction

Colleges and universities were forced to close their doors in March 2020 as community spread of COVID-19 increased across the United States.^1^ With school closure, some students at historically Black colleges and universities (HBCUs) faced challenges related to housing insecurity and inadequate internet access. In March 2020, when Delaware State University (DSU) was forced to close its campus, 200 students who were otherwise homeless without DSU remained on campus.^2^ While many schools struggled with reopening strategies for fall 2020, for HBCUs, the pandemic posed an existential threat to the education of their students, the schools’ financial sustainability, and the safety and security of their students who were at greatest risk of homelessness, food insecurity, and limited access to internet to continue online studies.^3^

Fall reopening of residential colleges and universities posed many challenges associated with shared living, bathroom, and dining facilities; the proclivity of young adults to socialize; and intimate classroom settings. In the absence of a vaccine, a multifaceted approach to decreasing communicability and preventing exponential increase in positivity was paramount. When planning for the fall semester during spring and summer 2020, many regions faced limited access to COVID-19 testing resources, and clarity on the best testing technologies and standards for testing strategy were only beginning to evolve.^4,5,6,7^

At DSU, like at all colleges, student, faculty, and staff safety was of primary importance; in addition to infection prevention, providing secure internet access and housing and food security made reopening in some capacity absolutely necessary. Prior to campus reopening, DSU developed protocols based on best available evidence with a goal of mitigating the outcomes associated with COVID-19. The objectives of this retrospective review were to investigate the association with with prevalence of disease spread of a comprehensive campus education program about the importance of shared responsibility for campus safety, describe the implementation of a frequent testing program, evaluate adequacy of quarantine and isolation dormitory capacity, and compare campus positivity rates with state rates. We hypothesized that DSU’s multifaceted approach to campus safety would be associated with a decrease in COVID-19 positivity rates compared with the surrounding community.

## Methods

This cohort study followed the Strengthening the Reporting of Observational Studies in Epidemiology (STROBE) reporting guideline. The study was determined to be exempt from review and informed consent by the Delaware State University Institutional Review Board (Common Rule, Category 4).

### Messaging and Nontesting Safety Measures

DSU’s messaging about campus safety began during early summer 2020. This included information regarding the planned testing program and specifically messaging that testing was “necessary, but not sufficient” to minimize spread of COVID-19.^8^ Messaging was delivered at least weekly to reinforce guidance from the US Centers for Disease Control and Prevention (CDC) and the Delaware Division of Public Health (DPH). Means of communication included web-based information, email and text blasts, signage, and videos about masking, social distancing, and handwashing. Additional data-focused communications were delivered based on changes in campus and community positivity rates and around important calendar events.

Multiple campus modifications were used to allow reopening associated with mitigated spread of disease. Residence hall census was decreased to 75%. Food service was converted to take away service (ie, grab and go). Masking was required at all times indoors and outdoors except in individuals’ residences. Nonresident entry to campus required daily screening using the CampusShield app (911Cellular).^9^ Learning was transitioned to a hybrid of in-person and virtual education. Classroom analytics were performed to limit classrooms to one-third capacity. Each room and building was evaluated by Academic Operations and the University COVID Task Force to review capacity and ventilation. Athletic competitions were canceled for the fall semester, but training continued with modifications consistent with state and federal recommendations.

### Testing Program

A twice-weekly polymerase chain reaction (PCR) testing program for all students, faculty, and staff was used. Predictable lab capacity with 24-hour turnaround of results was sourced through Guardant Health. At program inception, tests were performed according to US Food and Drug Administration (FDA) laboratory-developed test guidelines. The test, Guardant-19, was granted FDA emergency use authorization (EUA) on August 21, 2020.^10^ In December 2020, DSU plans were completed to develop a molecular diagnostics laboratory, and beginning in January 2021, specimens were transitioned to SalivaDirect (Yale School of Public Health) testing within the DSU laboratory.^11,12^

Rosters were developed to include individuals with a presence of 15 minutes per day on campus, including faculty members, resident and commuter students, and third-party vendors. Results tracking systems and test sites were developed, and test site personnel were trained. Test sites were run by students and faculty. Specimens were self-collected anterior nasal swab or saliva samples. Samples were returned in bins, maintained within the temperature limits of the media, for transportation to the laboratory. Students with potential COVID-19 symptoms were instructed to contact Student Health Services (SHS) for telehealth evaluation. If symptoms warranted testing, these students were placed in quarantine, test supplies were delivered, and students were moved to isolation if testing positive or further treated based on clinician judgment.

COVID-19 tests were first administered on July 9, 2020, with initial sessions allowing for validation of workflow and user interfaces. After validation, plans for whole campus screening were developed. This study evaluates the screening performed from August 16, 2020, to April 30, 2021.

### Results Delivery and Action Plan

COVID-19 results were delivered electronically to the individual, director of SHS, and DPH with a target turnaround time of 36 hours. Positive results were communicated directly to the individual by SHS, which also performed contact tracing per DPH and CDC guidelines. Nonresident individuals with positive results were instructed to isolate at home per CDC guidelines. Resident students who tested positive were housed in isolation residence halls or had the option to go home, and their close contacts were housed in quarantine facilities or had the option to go home. The maximum available occupancy in the quarantine and isolation residence halls was 53 individuals. Medical care, educational resources, and food services were provided for students within these facilities. Individuals with positive results were then removed from the testing roster for 90 days.

### Statistical Analysis

Test-taker rosters were continually tracked as the campus population fluctuated during the academic year. Total specimens collected, positive and negative results, and quarantine and isolation residence hall occupancy levels were tracked weekly. Symptoms and disease severity were tracked via interviews with individuals conducted by SHS nursing staff. Compliance rate, defined as total tests per on-campus population, was evaluated retrospectively. Percent positivity was retrospectively compared with statewide percent positivity and case rates according to reporting by DPH.^13^ Week over week increases in positivity were analyzed for association with school breaks, holidays, and major dates using R statistical software version 4.0.5 (R Project for Statistical Computing). Within the R package ggplot2, 2-sided tests were used to assess significant differences between positivity rates by week, with significance set at *P* < .05. Outcome measures included cumulative tests, infections, daily quarantine and isolation residence hall occupancy, and comparison with statewide COVID-19 positivity rates obtained from published DPH data. Data were analyzed from July through September 2021.

## Results

Enrollment for 2020 to 2021 fall and spring semesters was 5027 students and 4679 students, respectively, vs 5054 students and 4541 students for fall and spring 2019 to 2020. There were 1575 residential students in the fall and 1450 residential students in the spring, compared with 2200 residential students in the fall and spring of academic year 2019 to 2020. There were 415 nonresident students with on-campus classes and 330 faculty and staff members with significant on-campus presence during the academic year. The campus testing cohort included 2320 individuals (1575 resident students, 415 nonresident students, and 330 faculty and staff members). There were 1488 (64.1%) women and 832 (35.9%) men; mean (SD) age was 27.5 (12.9) years. There were challenges with roster development and management, given that individuals may have fallen into multiple categories (eg, graduate students as students and faculty) or changed categories (eg, faculty or students changing status from on-campus status to remote or vice versa).

Campus opened to resident students in the fall during the week of August 16, 2020, with most students leaving campus on November 24, 2020. After that date, 225 student athletes and students with housing insecurity remained on campus. Campus reopened for the spring semester during the week of January 4, 2021, and the semester ended during the week of April 26, 2021.

During the fall semester, 36 500 tests were administered. Prior to Thanksgiving, 33 034 tests (weekly range: 1245-3274 tests; mean [SD], 2359 [602] tests) were administered, and after Thanksgiving, 3466 tests (weekly range, 120-885 tests; mean [SD], 577 [260] tests) were administered. During the spring semester, 39 045 tests were administered, and during the week of January 4, 2021, 900 tests were administered. From January 11 to April 30, 2021, there were 38 145 tests administered (weekly range, 1981-2675 tests; mean [SD]; 2384 [240] tests) (Table 1).

**Table 1.** COVID-19 Tests Administered 2020 to 2021

Testing window	Individuals tested, No.			Positivity rate, %	Statewide positivity rate per 100 000 individuals/d
	Total tests	With negative results	With positive results		
8/16/20-8/22/20	1584	1571	13	0.82	7.5
8/23/20-8/29/20	1245	1237	8	0.64	9.4
8/30/20-9/5/20	2500	2486	14	0.56	10.8
9/6/20-9/12/20	2680	2662	18	0.67	10.2
9/13/20-9/19/20	2919	2907	12	0.41	10.6
9/20/20-9/26/20	3274	3265	9	0.27	10.5
9/27/20-10/3/20	2687	2685	2	0.07	14.3
10/4/20- 10/10/20	3016	3015	1	0.03	14.1
10/11/20- 10/17/20	2869	2866	3	0.10	14.5
10/18/20-10/24/20	2724	2723	1	0.04	12.9
10/25/20-10/31/20	1854	1852	2	0.11	17.3
11/1/20-11/7/20	1668	1646	22	1.32	21.7
11/8/20- 11/14/20	2159	2136	23	1.07	33
11/15/20-11/21/20	1855	1833	22	1.19	45.5
11/22/20-11/28/20	589	582	7	1.19	51.2
11/29/20-12/5/20	885	869	16	1.81	70
12/6/20-12/12/20	758	751	7	0.92	84.4
12/13/20-12/19/20	742	736	6	0.81	80
12/20/20-12/26/20	372	372	0	0.00	68.3
12/27/20-1/2/21	120	118	2	1.67	68.4
1/4/21-1/8/21	900	864	36	4.00	84.0
1/11/21-1/15/21	1981	1968	13	0.66	76.5
1/18/21-1/22/21	2549	2540	9	0.35	69.6
1/25/21-1/29/21	2542	2534	8	0.31	55
2/1/21-2/5/21	2383	2373	10	0.42	39.8
2/8/21-2/12/21	2315	2284	31	1.34	40.1
2/15/21-2/19/21	2179	2164	15	0.69	28.7
2/22/21-2/26/21	2514	2495	19	0.76	29.9
3/1/21-3/5/21	2638	2620	18	0.68	25.1
3/5/21-3/12/21	2507	2495	12	0.48	20.9
3/15/21-3/19/21	2516	2508	8	0.32	21.6
3/22/21-3/26/21	2675	2669	6	0.22	25.9
3/29/21-4/1/21	2028	2024	4	0.20	33.8
4/5/21-4/9/21	2090	2082	8	0.38	32.3
4/12/21-4/16/21	2602	2588	14	0.54	37.3
4/19/21-4/23/21	2624	2588	36	1.37	34.5
4/26/21-4/30/21	2002	1971	31	1.55	26.5

Given a population maximum of 2320 individuals in fall and 2195 individuals in spring requiring participation in the twice-weekly testing program and the previously given totals for tests administered, the testing compliance minimum was a mean (SD) of 51.2% (10.7%) and 54.3% (5.5%) in the fall and spring semesters, respectively. There was a component of testing population loss associated with individuals transitioning out of the testing program (eg, faculty members and commuters no longer remaining present on campus, resident students leaving for extended durations, and prior positive results), but the population decrease could not be accurately measured.

There were 168 unduplicated positive COVID-19 test results (weekly range, 0-23 results) during the fall semester and 267 unduplicated positive test results (weekly range, 4-36 results) in the spring semester. There were 36 312 negative results in the fall semester and 38 767 negative results in the spring semester. Positivity rates ranged from 0 of 372 tests to 16 of 869 tests (1.8%) in the fall semester (mean [SD] positivity rate, 0.5% [0.5%]) and 4 of 2028 tests (0.2%) to 36 of 900 tests (4.0%) in the spring semester (mean [SD] positivity rate, 0.8% [0.9%]). When entry testing for the spring semester was excluded, the spring positivity rates ranged from 4 of 2024 tests (0.2%) to 31 of 1971 tests (1.6%) (Table 1). Of 168 individuals with positive test results in the fall semester, there were 131 students and 37 faculty or staff members. Of 267 individuals with positive test results in the spring semester, there were 220 students and 47 faculty or staff members. Data comparing positivity rates of resident vs commuter students were not reliable given that residency status changed during the semester for many students. In the fall and spring, there were 20 and 11 duplicate positive test results, respectively, after individuals were errantly retested within 90 days of initial positive results.

Of 168 individuals with positive test results in the fall semester, 127 individuals stayed asymptomatic. Of 267 individuals with positive test results in the spring semester, 153 individuals stayed asymptomatic. There were no hospitalizations or deaths. Of 41 individuals who were symptomatic the fall, there were 36 students and 5 employees. Of 114 individuals who were symptomatic in the spring, there were 86 students and 28 employees (Table 2).

**Table 2.** Positive Test Results by Symptomatic vs Asymptomatic Infection

COVID-19 test results	Positive test results		
	Total, No.	No. (%)	
		Symptomatic infection	Asymptomatic infection
**Fall**			
Students	131	36 (27.5)	95 (72.5)
Faculty and staff	37	5 (13.5)	32 (86.5)
Total	168	41 (24.4)	127 (75.6)
**Spring**			
Students	220	86 (39.1)	134 (60.9)
Faculty and staff	47	28 (59.6)	19 (40.4)
Total	267	114 (42.7)	153 (57.3)

COVID-19 positivity rates for the State of Delaware ranged from 589 of 25 120 tests (2.3%) to 5405 of 54 596 tests (9.9%) during the fall semester (mean [SD] positivity rate, 4.8% [2.6%]) and 1336 of 37 254 tests (3.6%) to 3630 of 42 458 tests (8.5%) during the spring semester (mean [SD] positivity rate, 5.1% [1.3%]). Positivity rates on DSU campus were mean (SD) 4.4 (2.6) percentage points lower than statewide rates during the fall semester (*P* < .001) and 5.6 (1.6) percentage points lower during the spring semester (*P* < .001) (Figure). In R analysis, there were no statistically significant differences week to week with an α level equaling .05. The overall positivity rate during the entire academic year was 466 positive tests among 75 545 total tests (0.62%).

**Figure.**
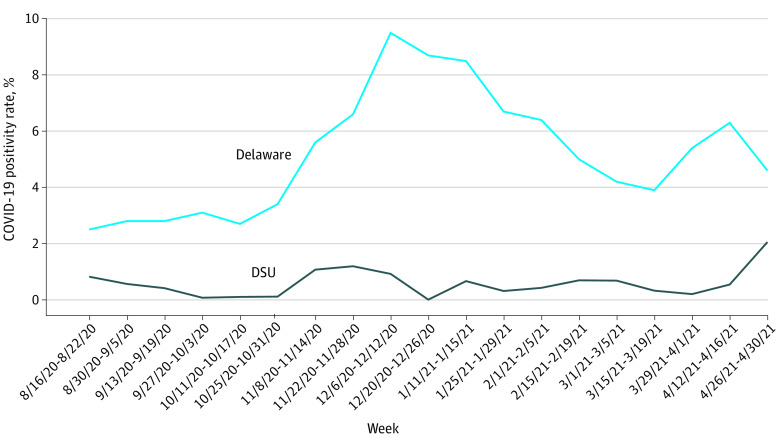
COVID-19 Positivity Rates at Delaware State University (DSU) vs State of Delaware

Individuals with positive test results were notified by telephone and required to self-report to isolate; close contacts were required to quarantine. Upon the individual’s arrival in the isolation dormitory (private room and bathroom), written instructions were provided regarding meals, length of stay (based on CDC guidance), and contact information for student health and the isolation resident hall supervisor. Individuals could isolate or quarantine on campus or off campus (ie, at home), although students were encouraged to isolate or quarantine on campus. In the fall semester, based on COVID-19 test results, 200 individuals required quarantine (46 individuals on campus and 154 individuals off campus) and 168 individuals required isolation (67 individuals on campus and 101 individuals off campus). In the spring semester, 202 individuals required quarantine (87 individuals on campus and 115 individuals off campus) and 278 individuals required isolation (87 individuals on campus and 191 individuals off campus). The maximum occupancy in isolation and quarantine dormitories was 47 individuals. Total daily quarantine and isolation residence hall occupancy ranged from 0 to 43 students in the fall and from 1 to 47 students during the spring. Roommates and other close contacts were identified through contact tracing and were quarantined for 14 days or moved to isolation if they subsequently tested positive.

## Discussion

In March of 2020, the COVID-19 pandemic caused a secondary academic crisis. US colleges and universities closed their campuses. At DSU, 200 students were allowed to stay on campus because, without university housing, they were otherwise homeless.^2^ These students served as a proxy for the socioeconomic challenges faced by many other HBCU students. Reopening in fall 2020 was imperative to meet the academic and basic needs of the DSU student body. Reopening was also financially imperative. Mean HBCU endowments are one-eighth those of historically predominantly white colleges and universities. The lost revenue from a failure to reopen posed an existential threat to many HBCUs.^14^

HBCUs serve a critical need for the United States. More than half of students at HBCUs are first-generation college students, more than 70% are from low-income families or are Pell Grant awardees, and more 90% are awarded some type of financial aid. Additionally, while 9% of Black college students attend HBCUs, among Black individuals, 40% of engineers, 50% of lawyers, and 70% of physicians and dentists earned degrees at HBCUs.^14^ These institutions faced an increased risk when reopening given that infection risks and comorbidity among Black students and other students with increased socioeconomic risks students exceed those among an aged-matched general population. In Delaware, COVID-19 infection rates among individuals self-identifying as Black was 46% higher than among individuals self-identifying as White.^13^ Furthermore, evidence suggests increased comorbidity and more severe complications associated with COVID-19 among Black individuals.^15,16^ Additionally, in the absence of a robust testing program, the risks of resident students with asymptomatic infection returning home on weekends or holidays to urban areas or to multigenerational homes posed a significant risk of introducing infection to close-knit communities.

In the period analyzed in this cohort study, DSU sought to implement a strategy of overlapping safety mechanisms associated with maximized safety during campus reopening. Robust testing was a critical component to this strategy. Not only did the testing aid in identification of asymptomatic infection, but test sites were another opportunity to engage in messaging regarding COVID-19 mitigation. Mass test sites were strategically placed to be a focal point of campus life. Frequent messaging through videos, signage, emails, texts, and social media were used to provide updates and to encourage a mission of shared responsibility.

DSU stayed open during the academic year, providing academic and basic needs security to the campus population. There were no serious illnesses and no deaths. The isolation and quarantine residence halls maintained a manageable census, and there was no exponential increase in positivity. While the close of the spring semester did see an increase in positivity rates, these individuals knew of their infection status and were encouraged to reside on campus to avoid introducing infection into home communities.

This study’s findings suggest that the aims of the DSU’s multifaceted approach to campus safety met the prescribed goals of a campus reopening associated with minimal morbidity in the school, local, and student home communities while meeting the academic needs of the student body and maintaining the fiscal stability of the institution. However, execution of the testing program presented many challenges. DSU partnered with Testing for America (TFA), a national nonprofit, to receive assistance in testing program development. Together, DSU and TFA posited that twice-weekly PCR testing was preferred for workflow and accuracy over antigen testing.^17,18^ Guardant Health was identified as a vendor that had produced a lab-developed test pending EUA. By partnering with GH prior to EUA, DSU was able to secure lab capacity in the setting of regional and national testing shortages. With TFA’s assistance, DSU was also fortunate to identify philanthropic support to cover the entire cost of the testing program. Alleviation of this financial stress was a prerequisite for successful program execution. In late fall 2020, DSU began developing a molecular diagnostics laboratory that performed its first on-campus COVID-19 testing using SalivaDirect in December 2020. By March 2021, the lab had reached capacity to perform all COVID-19 testing for the campus population. The cost of laboratory development was funded through a local government grant, significantly decreasing the cost per test and decreasing turnaround time to less than 24 hours.^11^

Throughout the academic year, compliance with testing was challenging. Consistent patterns in noncompliance could not be identified based on residence hall, year of study, sex, or faculty vs student status. The only group for whom compliance rate could be measured accurately consisted of student athletes, coaches, and training staff, among whom compliance neared 100%. Tracking of athletes was accomplished by linking participation in athletics to compliance with testing. However, because most classes were hybrid or virtual, there was not an equivalent mechanism for linking testing compliance to participation in the academic program.

In fall 2021, DSU will mandate vaccination of all students, and vaccination of faculty and staff will be strongly encouraged. Given the high efficacy of vaccination, the value of a frequent testing program moving forward requires further study. Increased penetration of COVID-19 variants, potential for waning immunity, and breakthrough cases all suggest that some form of continuous testing is needed. DSU plans to continue weekly testing for vaccinated individuals and twice-weekly testing for unvaccinated individuals. Based on current levels of disease penetration, mask mandates will continue, but an increased number of educational opportunities will be offered in person. While the cost-benefit analysis of a frequent testing program in the setting of high vaccination rates and low community positivity may favor less testing, the development of the DSU molecular testing lab lowers cost per test significantly.

### Limitations

We acknowledge that this study has several limitations. First, we have no comparative experiences with testing at a lower frequency or with antigen tests in a similar population. Mathematical modeling suggests that with frequent testing, lower-sensitivity tests are adequate. However, given the lower than anticipated compliance rate in the DSU campus population, the selection of high sensitivity PCR tests was serendipitous.^17^ Furthermore, there were challenges with test taker roster management and compliance. We acknowledge that universal compliance would more accurately measure whole campus positivity rate.

## Conclusions

DSU was able to reopen its campus for the 2020 to 2021 academic year using a multifaceted approach to campus safety. This allowed DSU to provide for the academic and basic needs of its campus community. The use of a combination of in-person, hybrid, and online classes; multi-media messaging; and a frequent testing program was associated with a 0.6% positivity rate and no hospitalizations or deaths. These findings suggest that further study of the cost-benefit rationale of a frequent testing program in the setting of a fully vaccinated population is warranted.
